# Relationship between small dense low-density lipoprotein cholesterol with carotid plaque in Chinese individuals with abnormal carotid artery intima-media thickness

**DOI:** 10.1186/s12872-021-02023-4

**Published:** 2021-04-27

**Authors:** Fang Liu, Zheng Wang, Xia Cao, Yingxia Pan, Erqiang Zhang, Jiahuan Zhou, Lina Zheng

**Affiliations:** 1 Health Management Centre, Kaifeng Central Hospital, Kaifeng, 475000 Henan China; 2 Shanghai Zhangjiang Institue of Medical Innovation, Shanghai Biotecan Pharmaceuticals Co., Ltd, Shanghai, 201204 China

**Keywords:** Small dense low-density lipoprotein cholesterol, Intima media thickness, Carotid plaque, Atherosclerosis

## Abstract

**Aim:**

To investigate the relationship of small dense low-density lipoprotein cholesterol (sdLDL-C) to carotid artery intima-media thickness (CA-IMT) and carotid plaque (CAP) in Chinese general population, and to evaluate whether sdLDL-C could be an independent risk factor for individuals with subclinical atherosclerosis.

**Methods:**

A total of 729 subjects were randomly collected from consecutive individuals from April 2019 to April 2020 for an annual health checkup. CA-IMT > 1.0 mm was defined as abnormal IMT. Plaque stability was measured by ultrasound examination based on the property of the echo. And sdLDL-C levels were detected by LipoPrint system. Multivariate logistic regression analysis was performed to identify factors associated with CA-IMT and carotid plaque.

**Results:**

The abnormal IMT group had significantly higher sdLDL-C levels than control group (*p* < 0.0001). And sdLDL-C levels were significantly positively correlated with IMT value (r = 0.1396, *p* = 0.0021) and presence of carotid plaque (r = 0.14, *p* = 0.002) in the subjects with abnormal IMT. In addition, subjects with higher levels of sdLDL-C (r = 0.11, *p* = 0.035) tended to have unstable CAP. After adjustment for age, gender and blood glucose, sdLDL-C level was an independent risk factor of the presence of CAP (OR = 1.59, 95% CI: 1.02–1.83, *p* = 0.034) in subjects with abnormal IMT.

**Conclusion:**

SdLDL-C is an independent risk factor of the occurrence of CAP in the Chinese subjects with abnormal IMT. Our findings provide supporting evidence that sdLDL-C might be an alternative way to predict CVD in early stage.

## Background

Cardiovascular disease (CVD) is a leading cause of early death and disability around the world [1]. Earlier identification of high CVD-risk individuals is of vital importance to reduce the mortality associated with CVD. Carotid artery intima-media thickness (CA-IMT) serves as one of the key markers for CVD in different populations [2, 3]. In addition, plaque composition and its instability in the carotid arteries is closely related to the risk for CVD [4, 5]. Early detection of vulnerable plaques is deemed to be an important factor in the prevention of CVD. Carotid ultrasonography is the major imaging modality for evaluating carotid wall thickness and providing information on plaque composition [6]. However, it is not a routine test for annual health check-up, especially in rural area. Thereby, exploration of potential biomarkers related to CA-IMT and carotid atherosclerotic plaque might be an alternative way to predict CVD in early stage.

Low–density lipoprotein cholesterol (LDL-C) is a well-studied risk factor of CVD [7, 8]. While growing evidence has challenged the conventional view of LDL-C, as a large part of patients with normal plasma LDL-C levels still experienced CV events [9]. LDL consists of seven subclasses (LDL 1—LDL 7), LDL 1 and 2 are defined as larger-buoyant LDL, and LDL 3 to 7 are defined as small-dense (sd) LDLs according to their size and density [10]. Notably, sdLDL-C is thought to be more atherogenic. Evaluated level of sdLDL-C has been reported to be correlated with an increased risk of CVD, and sdLDL-C is considered to be a more suitable predictor for cardiovascular disease outcomes than total LDL-C level [11]. More studies trend to evaluate the treatment effect on the changes in sdLDL-C level other than total LDL-C level [12, 13]. Since evidence showed that patients who took lipid-lowering drugs and reached the set LDL-C level were still at risk of cardiovascular and cerebrovascular events [13, 14]. Previous studies related to the sdLDL-C mainly focused on the patients who have experienced cardiovascular and cerebrovascular events [15, 16]. For instance, studies have shown that sdLDL-C is an independent risk factor for IMT in acute ischemic stroke [17], as well as for unstable plaques in acute cerebral infarction (ACI) patients [18]. While, atherosclerosis progresses silently. By the time symptoms do arise, it’s usually advanced and serious. sdLDL-C has been found playing a role in the plaque progression. The levels of sdLDL-C are positively correlated with the severity of carotid stenosis in cerebral infarction patients [19]. And cholesterol in the dense LDL fractions was found significantly affect carotid plaque cellular composition in patients with severe carotid artery stenosis [20]. However, study related to the association of sdLDL-C with CA-IMT and carotid plaque in individuals with clinically silent atherosclerosis are relatively sparse.

According to the report on cardiovascular disease in China, 40% of deaths in the Chinese population were due to CVD [21]. This study investigated the relationship of sdLDL-C to CA-IMT and carotid plaque in Chinese general population, especially explore the association of sdLDL-C with carotid plaque (CAP) stability in the subjects with abnormal IMT via a rapid, quantitative method for LDL subfractionation assay. Whether sdLDL-C could be an independent risk factor for individuals with subclinical atherosclerosis was also evaluated.

## Methods

### Study participants

Seven hundred and twenty-nine subjects were randomly collected from consecutive individuals visiting the Kaifeng Central Hospital for an annual health checkup from April 2019 to April 2020. The including criteria: (1) native Chinese; (2) age ≥ 14 years old; (3) underwent carotid ultrasound. And the exclusion criteria: (1) subjects with a history of CVD (myocardial infarction, angina pectoris, and stroke); (2) treatment with any medications that could influence lipid; (3) unwillingness to participate. The subjects received oral information before they gave their consent to participate. The study was approved by the Ethics Committee of Kaifeng Central Hospital.

### Carotid ultrasonographic examination

Carotid artery measurements were performed with high-resolution color Doppler ultrasound (DC-70; MINDRAY, Shenzhen, China) with a 7.5 MHz annular array probe (Toshiba PLT-704SBT). cIMT protocol followed the Mannheim Carotid Intima-Media Thicknessand Plaque Consensus (2004–2006–2011) [22]. Both the left and right carotid arteries were scanned by a trained operator with ten years of experience. The imaging protocol involved obtaining a single longitudinal lateral view of the distal 10 mm of the right and left common carotid arteries (CCAs) and 3 longitudinal views (anterior-oblique, lateral, and posterior-oblique) of each internal carotid artery (ICA) [23, 24]. The distance between the leading edge of lumen-intima interface and the leading edge of the media-adventitia interface was recorded as IMT [25]. And the larger IMT of the bilateral common carotid artery was used for analysis in this study. According to data of healthy Chinese subjects [26], abnormal IMT was defined as a value over 1.0 mm. The determination of plaque stability was based on the character of echo in ultrasound. Low-level or equal-level echo indicated unstable plaque, while high-level echo indicated stable plaque [17].

### Laboratory tests

Blood glucose, total cholesterol, triglyceride, and lipoproteins were analyzed from fasting blood samples. And above parameters were determined by using an automatic analyser (XN-2000, SYSMEX, Kobe, Japan). The plasma sdLDL-C assessment was performed by LipoPrint system (Quantimetrix Corp., CA, USA), which is the FDA-cleared diagnostic test for analyzing cholesterol of all LDL subfractions (1–7). The Lipoprint LDL Kit consists of precast, high resolution polyacrylamide gel tubes, a loading gel solution containing a lipophilic dye and the buffer salts. The dye binds proportionally to the relative amount of cholesterol in each lipoprotein. The prestained lipoproteins subsequently undergo electrophoresis. As the lipoprotein particles migrate through the separating gelmatrix, they are resolved into lipoprotein bands according to their particle sizes from largest to smallest due to the sieving action of the gel: high-density lipoprotein (HDL) migrates the farthest, followed by small dense LDL, larger-buoyant LDL, Midbands (comprising primarily intermediate density lipoprotein) and very low density lipoprotein [27]. The electrophoresed gels are analyzed with Lipoware, a configured software that calculates the levels of cholesterol in each subfraction. A color coded profile is generated for ease of interpretation.

### Statistical analysis

All tests were performed by SPSS program version 19.0. Continuous data are presented as median (minimal and maximal values), and categorical variables are shown as percentage (%). Pearson's correlation coefficient analyses were used to explore the relationships between CA-IMT values and sdLDL-C values and other clinical variables. The variables data follow a non-normal distribution via Kolmogorov–Smirnov Tests. The Mann–Whitney *U* test was used to compare the continuous data between two groups, and Kruslal-Wallis *H* test was used to analyze the differences among more than two groups. The Fisher’s exact test was employed to compare categorical data. Multivariate logistic regression analysis was performed to identify factors associated with CA-IMT and carotid plaque. Differences with *p* < 0.05 were considered to be statistically significant.

## Results

### Comparison between abnormal IMT group and control group

**Fig. 1:**
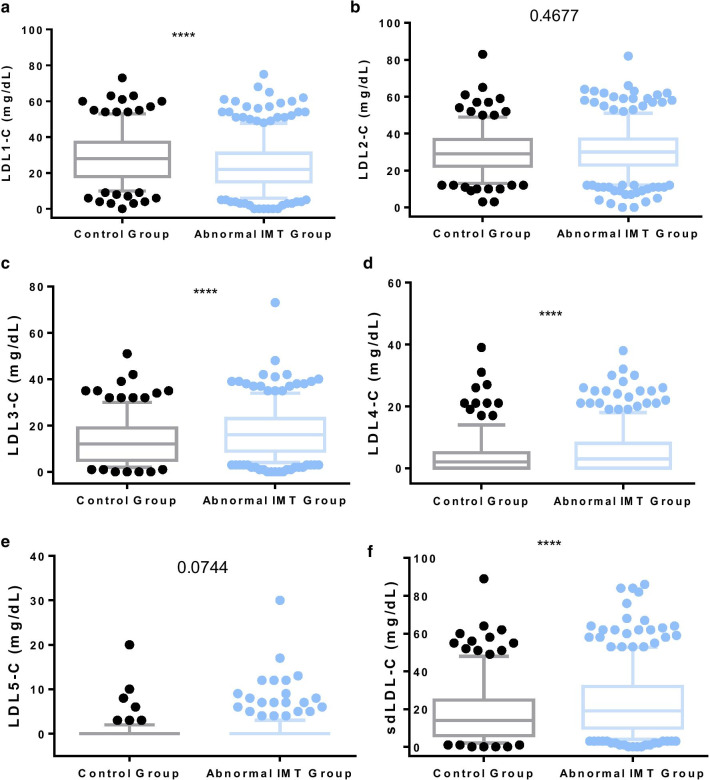
Comparisons between abnormal IMT group and control group in levels of the LDL1-5 -C and sdLDL-C (LDL3-5 -C)

Based on the cut-off value of IMT > 1.0 mm as abnormally increased carotid IMT, a total of 485 subjects were in the abnormal IMT group, and 244 subjects were in the control group. The median age of the control group and abnormal IMT group was 43 and 57, with significantly different (*p* < 0.0001). The percentage of males in abnormal IMT group was 72.2%, significantly higher than that of control group (*p* < 0.0001). In addition, There were significant differences in smoking and hypertension history between the two groups (*p* = 0.002 and *p* < 0.0001, respectively). The results showed that the abnormal IMT group had significantly higher BMI, blood pressure, blood glucose, triglyceride, sdLDL-C levels (especially LDL3-C and LDL4-C) than control group (*p* < 0.0001), and had significantly lower LDL1-C level than control group (*p* < 0.0001). While there was no significant difference in LDL-C level between abnormal IMT group and control group (*p* = 0.185). In addition, we also investigate the dietary habit of all the subjects. And we found that there was no significant difference in dietary favor and seafood consumption between the two groups (*p* = 0.239, *p* = 0.673) (Table 1). In this study, LDL6-C and LDL7-C were not detected in almost all of the subjects. Accordingly, these two variables were not included in the analysis. And the sdLDL-C level is the sum of LDL3-5 -C. Figure 1 shows the data distribution of the LDL1-5 -C and sdLDL-C (LDL3-5 -C) by Tukey method.

**Table 1:** Clinical features of all participants in the normal and abnormal IMT groups

Characteristics	Control group (IMT ≤ 1.0 mm)	Abnormal IMT group (IMT > 1.0 mm)	*p *value
	N = 244	N = 485	
Male gender (%)	49.6	72.2	** < 0.0001 **^**a**^
Age (years)	43 (16–72)	57 (29–89)	** < 0.0001 **^**b**^
BMI (kg/m^2^)	24.48 (17.30–36.68)	25.43 (18.83–41.21)	** < 0.0001 **^**b**^
Dietary favor (%)			0.239 ^**a**^
Well-balanced	43.0	46.8	
Preference for vegetarian	29.1	23.3	
Preference for meat	27.9	29.9	
Smoking (%)	8.6	17.1	**0.002**^**a**^
Hypertension history (%)	11.1	25.9	** < 0.0001**^**a**^
Systolic pressure (%)	121 (90–193)	132 (90–197)	** < 0.0001**^**b**^
Diastolic pressure (%)	75 (50–110)	81(45–155)	** < 0.0001**^**b**^
Seafood consumption (%)			0.673^**a**^
Seldom (< 1 time per week)	95.9	96.7	
Often (≥ 2 times per week)	4.1	3.3	
Fasting blood glucose (mmol/L)	5.30 (4.24–12.55)	5.62 (1.75–18.58)	** < 0.0001**^**b**^
Total cholesterol (mmol/L)	4.74 (2.71–7.94)	4.92 (2.24–8.94)	0.061^**b**^
Triglyceride (mmol/L)	1.21 (0.06–8.28)	1.49 (0.36–16.72)	** < 0.0001**^**b**^
HDL-C (mmol/L)	1.42 (0.67–6.40)	1.32 (0.51–4.50)	** < 0.0001**^**b**^
LDL-C (mmol/L)	2.69 (1.07–4.68)	2.83 (0.79–10.12)	0.185^**b**^
LDL1-C (mmol/L)	28 (0–73)	22 (0–75)	** < 0.0001**^**b**^
LDL2-C (mmol/L)	29 (3–83)	30 (0–82)	0.468
LDL3-C (mmol/L)	12 (0–51)	16 (0–73)	** < 0.0001**^**b**^
LDL4-C (mmol/L)	2 (0–39)	3 (0–38)	** < 0.0001**^**b**^
LDL5-C (mmol/L)	0 (0–20)	0 (0–30)	0.074
sdLDL-C (mmol/L)	14 (0–89)	19 (0–86)	** < 0.0001**^**b**^

The values in bold mean statistically significant

^a^Fisher’s exact test

^b^Mann-Whitney *U* test

### Correlation between clinical factors and CA-IMT

**Fig. 2:**
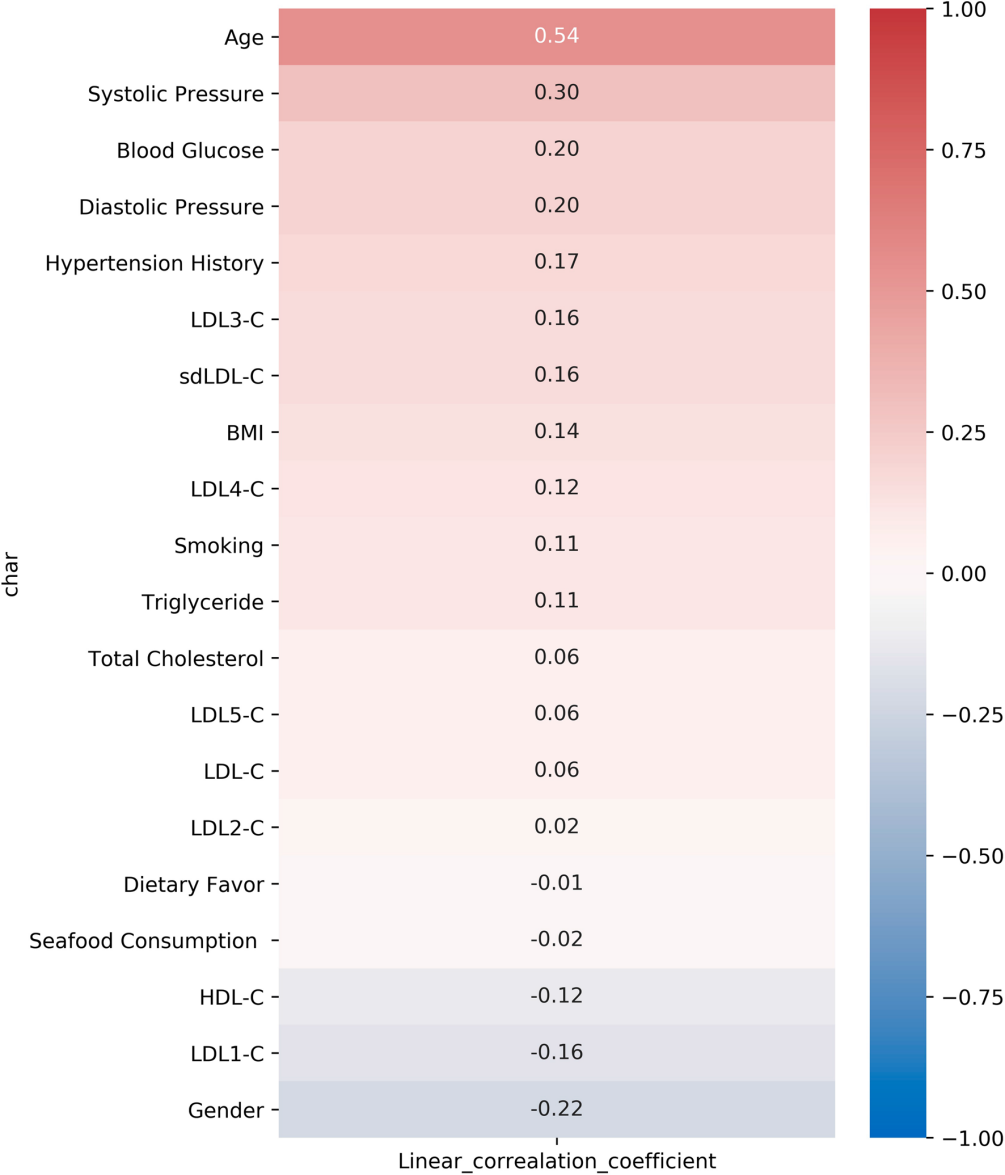
Pearson correlation between clinical characteristics and IMT

Pearson correlation analysis was conducted to determine the correlation of clinical risk factors and the presence of abnormal IMT (IMT > 1.0 mm) (Fig. 2). The results showed that sdLDL-C (r = 0.16, *p* < 0.0001), especially LDL3-C (r = 0.16, *p* < 0.0001) and LDL4-C (r = 0.12, *p* = 0.0012) were positively associated with abnormal IMT presence. In addition, age (r = 0.54, *p* < 0.0001), systolic pressure (r = 0.30, p < 0.0001), blood glucose (r = 0.20, *p* < 0.0001), diastolic pressure (r = 0.20, p < 0.0001), hypertension history (r = 0.17, p < 0.0001), BMI (r = 0.14, *p* < 0.0001), smoking (r = 0.11, p = 0.0020) and triglyceride (r = 0.11, *p* = 0.0023) were also found positively correlated with IMT abnormality. Meanwhile, there is a significant inverse correlation between abnormal IMT with the levels of HDL-C (r = -0.12, *p* = 0.001) and LDL1-C (r = -0.16, *p* < 0.0001). And males are more related with the presence of abnormal IMT (r = -0.22, *p* < 0.0001).

**Fig. 3:**
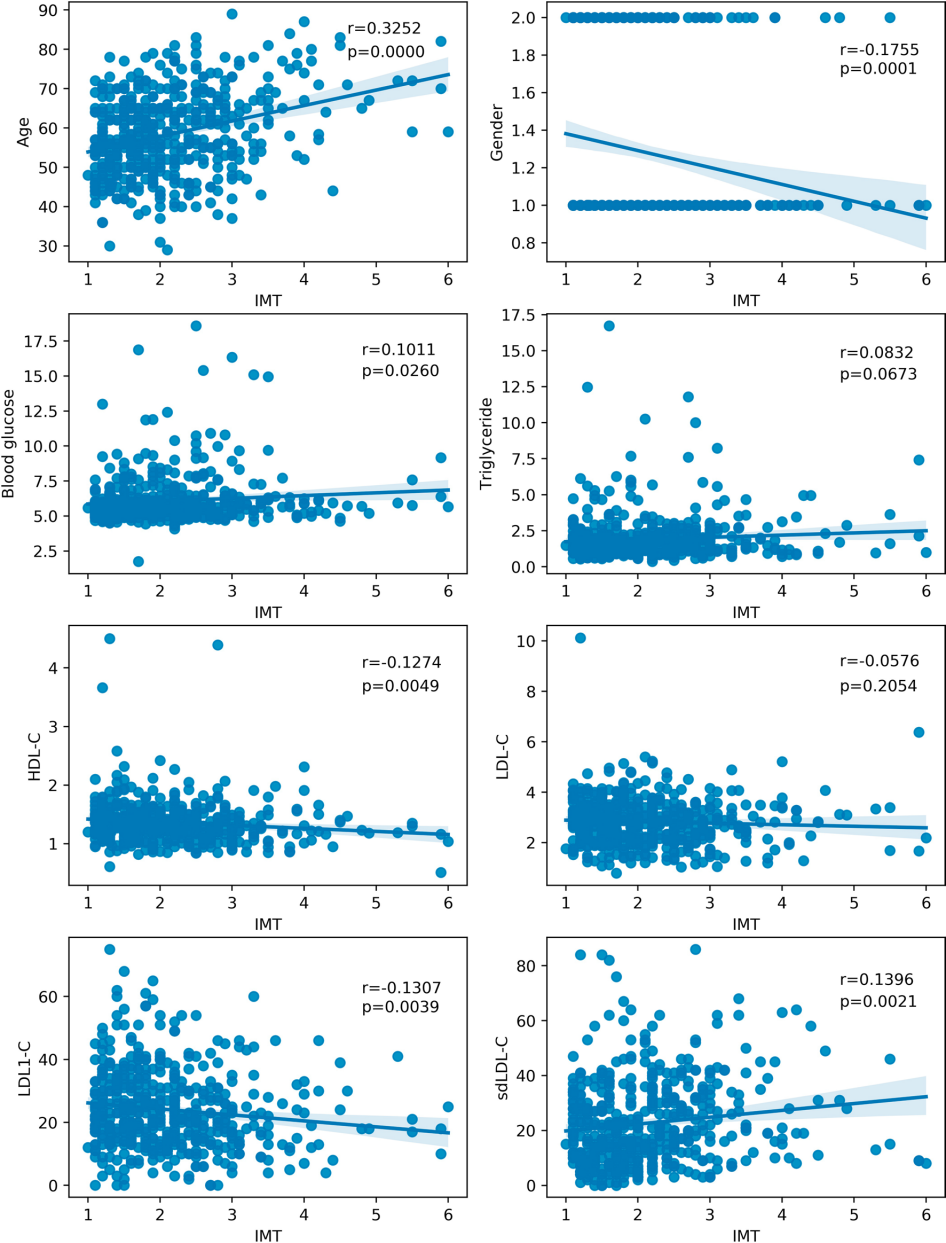
Pearson correlation analysis of IMT and other clinical factors in individuals with abnormal IMT

Furthermore, we intend to understand the relationship between sdLDL-C and CA-IMT in the individuals with clinically silent atherosclerosis. Thus, we analyze the correlation between clinical factors and CA-IMT value in the subjects with abnormal IMT. As shown in the Fig. 3, IMT was significantly positively correlated with the level of sdLDL-C (r = 0.1396, *p* = 0.0021), instead of LDL-C (r = 0.0576, *p* = 0.2054).

### Association of sdLDL-C with carotid plaque

We also investigated the association of sdLDL-C with carotid plaque (CAP) in the subjects with abnormal IMT. Of the 485 subjects with abnormal IMT, 371 subjects were with CAP. The data from Table 2 indicated that subjects with CAP were older (60 *vs* 53, *p* < 0.0001), consisted of more males (75.2% *vs* 62.3%, *p* = 0.009), with a higher ratio of having hypertension history (28.8% *vs* 16.7%, *p* = 0.010), and with higher systolic pressure (*p* = 0.0005) and glucose level (*p* = 0.003), comparing to the group without CAP. Plasma sdLDL-C levels (*p* = 0.005), including LDL3-C (*p* = 0.015), LDL4-C (*p* = 0.014) and LDL5-C (*p* = 0.003) were all significantly elevated in subjects with CAP (Table 2).

**Fig. 4:**
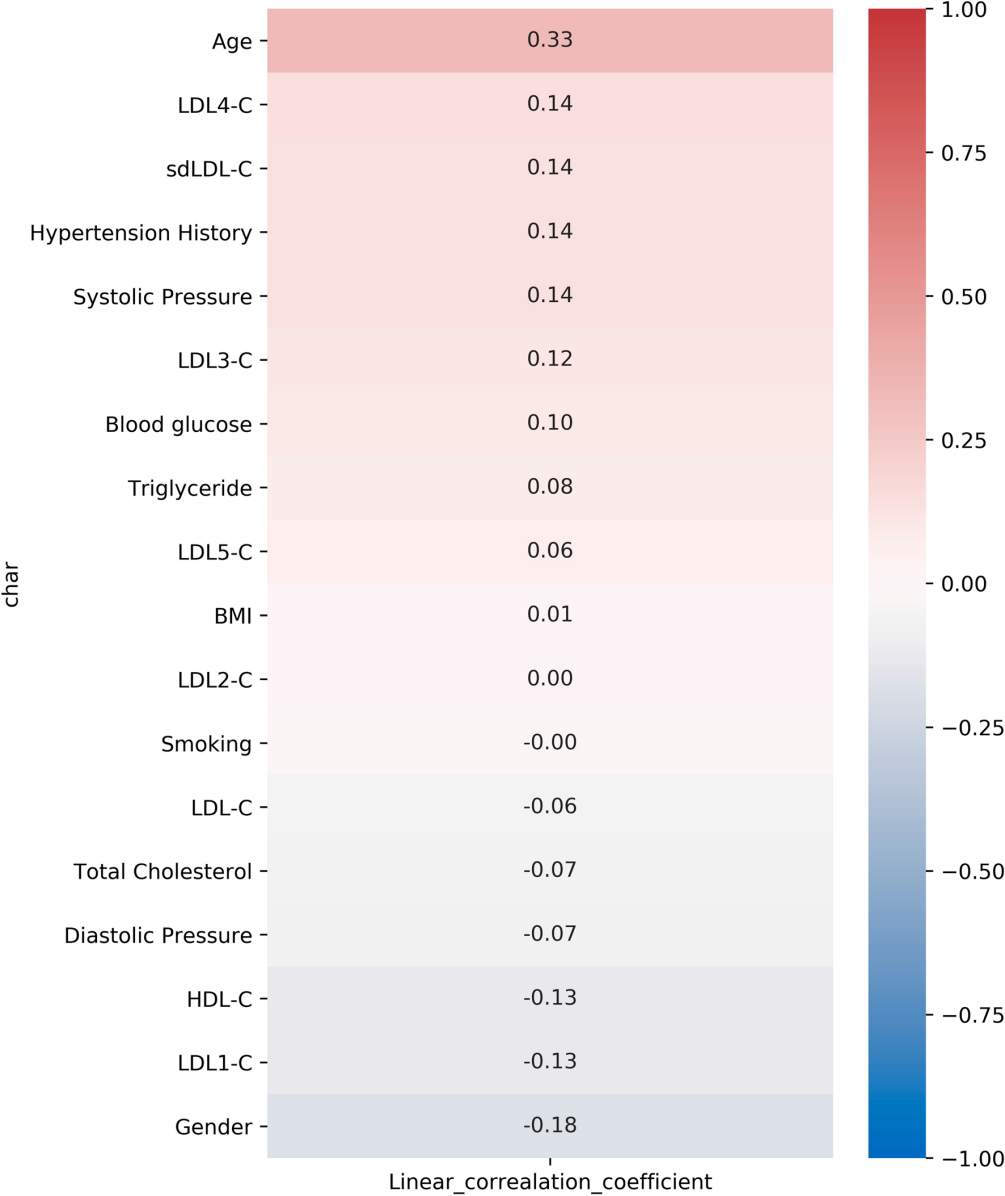
Pearson correlation analysis of CAP and clinical factors in individuals with abnormal IMT

Moreover, Pearson correlation analysis (Fig. 4) showed that sdLDL-C levels (r = 0.14, *p* = 0.002) were positively correlated with the occurrence of CAP. Additionally, data suggested that age (r = 0.33, *p* < 0.0001), hypertension history (r = 0.14, *p* = 0.0021), systolic pressure (r = 0.14, *p* = 0.0027) and blood glucose (r = 0.10, *p* = 0.026) were positively correlated with occurrence of CAP, and levels of HDL-C (r = − 0.13, *p* = 0.005) and LDL1-C (r = − 0.13, *p* = 0.004) were negatively correlated with it.

**Table 2:** Characteristics between the carotid plaque-free group and CAP group in individuals with abnormal IMT

Characteristics	Abnormal IMT without CAP	Abnormal IMT with CAP	*p *value
	N = 114	N = 371	
Male gender (%)	62.3	75.2	**0.009 **^**a**^
Age ( years)	53 (30–78)	60 (29–89)	** < 0.0001 **^**b**^
BMI (kg/m^2^)	25.43 (18.83–34.4)	25.43 (18.93–41.21)	0.701 ^b^
Smoking (%)	15.7	17.5	0.668 ^a^
Hypertension History (%)	16.7	28.8	**0.010 **^**a**^
Systolic Pressure (%)	127 (93–168)	134 (90–197)	**0.0005 **^**b**^
Fasting blood glucose (mmol/L))	5.44 (4.50–13.00)	5.68 (1.75–18.58)	**0.003 **^**b**^
Total Cholesterol (mmol/L)	5.02 (2.24–6.95)	4.84 (2.62–8.94)	0.130 ^b^
Triglyceride (mmol/L)	1.49 (0.53–12.46)	1.48 (0.36–16.72)	0.549 ^b^
HDL-C (mmol/L)	1.39 (0.61–4.50)	1.30 (0.51–4.39)	**0.007 **^**b**^
LDL-C (mmol/L)	2.93 (1.24–10.12)	2.77 (0.79–6.38)	0.092 ^b^
LDL1-C (mmol/L)	24 (0–75)	21 (0–68)	0.075 ^b^
LDL2-C (mmol/L)	30 (0–62)	30 (0–82)	0.747 ^b^
LDL3-C (mmol/L)	14 (0–34)	16 (0–73)	**0.015 **^**b**^
LDL4-C (mmol/L)	2 (0–26)	3 (0–38)	**0.014 **^**b**^
LDL5-C (mmol/L)	0 (0–30)	0 (0–17)	**0.003 **^**b**^
sdLDL-C (mmol/L)	16 (0–84)	20 (0–86)	**0.005 **^**b**^
IMT (mm)	1.2 (1.0–2.0)	2.2 (1.5–6.0)	** < 0.0001 **^**b**^

The values in bold mean statistically significant

^a^Fisher’s exact test

^b^Mann-Whitney *U* test

**Fig. 5:**
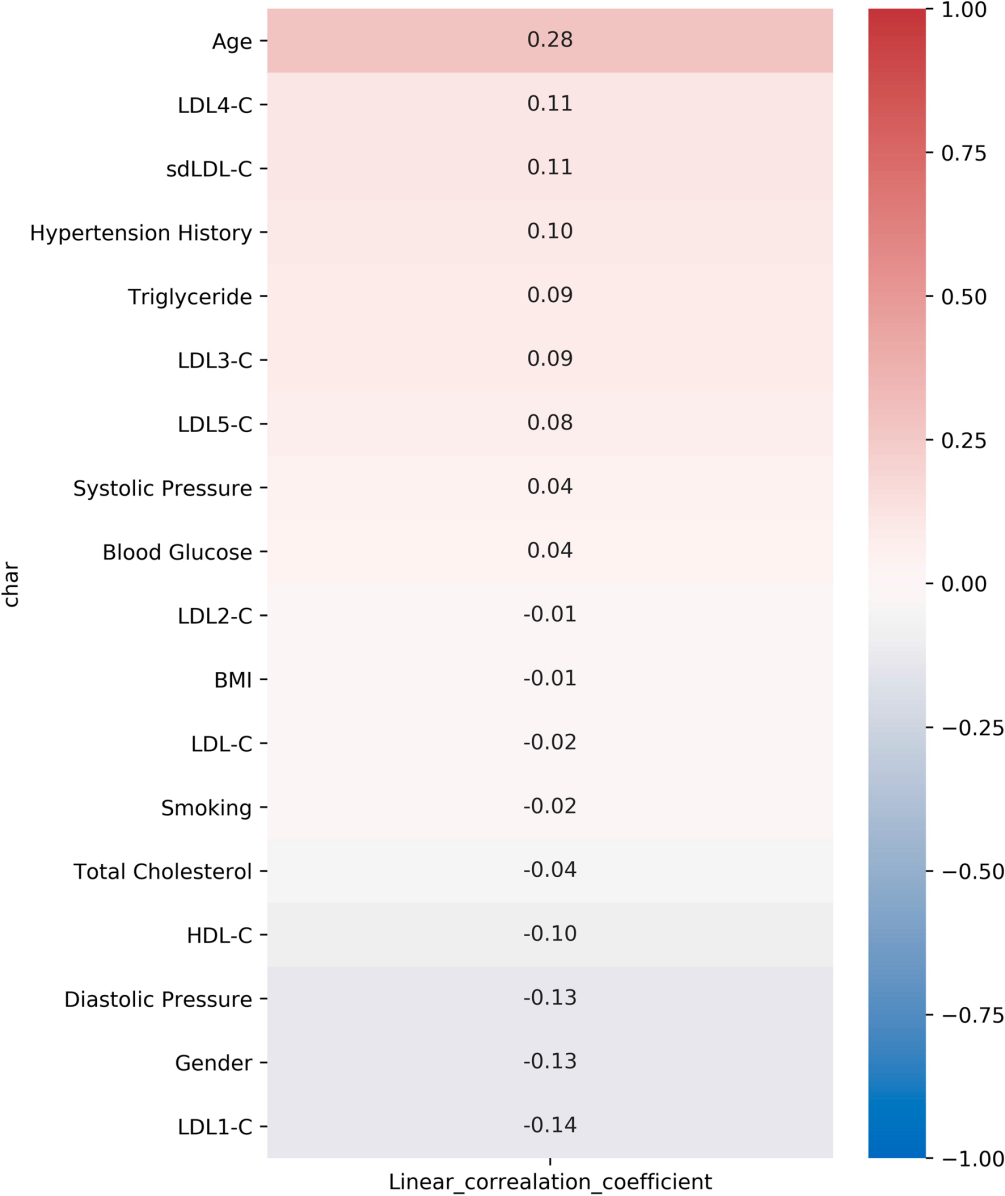
Pearson correlation analysis of CAP stability and clinical factors in individuals with abnormal IMT

In order to better understand the role of sdLDL-C in the individuals with asymptomatic atherosclerosis, we further analyze the relationship of sdLDL-C to carotid plaque stability. Of the 371 subjects with CAP, 343 subjects were with unstable CAP. The levels of sdLDL-C (*p* = 0.005), systolic pressure (*p* = 0.002) and blood glucose (*p* = 0.008) were highest in the subjects with unstable plaques, and the percentage of male (*p* = 0.024) and hypertension history (*p* = 0.035) was highest in the unstable CAP group. While the level of HDL-C (*p* = 0.026) was lowest in unstable CAP group, compared with other groups (Table 3. Moreover, the results obtained by the Pearson correlation analysis suggested that subjects with higher levels of sdLDL-C (r = 0.11, *p* = 0.035) tended to have unstable CAP (Fig. 5).

**Table 3:** Characteristics among the CAP-free, stable and unstable CAP groups in individuals with abnormal IMT

Characteristics	Abnormal IMT without CAP	Stable plaque	Unstable plaque	*p *value
	N = 114	N = 28	N = 343	
Male gender (%)	62.3	71.4	75.5	**0.024**^**a**^
Age (years)	53 (30–78)	61 (40–83)	60 (29–89)	** < 0.0001**^**b**^
BMI (kg/m^2^)	25.43 (18.83–34.4)	25.43 (19.22–34.26)	25.43 (18.93–41.21)	0.829^b^
Smoking (%)	15.7	16.9	25.0	0.502^**a**^
Hypertension history (%)	16.7	28.9	28.6	**0.035**^**a**^
Systolic pressure (%)	127 (93–168)	134 (90–179)	134 (92–197)	**0.002**^**b**^
Diastolic pressure (%)	81 (50–117)	80 (58–103)	81 (45–155)	0.903^b^
Fasting blood glucose (mmol/L)	5.44 (4.50–13.00)	5.59 (4.70–15.10)	5.69 (1.75–18.58)	**0.008**^**b**^
Total cholesterol (mmol/L)	5.02 (2.24–6.95)	4.69 (2.62–6.64)	4.88 (2.67–8.94)	0.124^b^
Triglyceride (mmol/L)	1.49 (0.53–12.46)	1.40 (0.44–8.23)	1.48 (0.36–16.72)	0.738^b^
HDL-C (mmol/L)	1.39 (0.61–4.50)	1.31 (0.9–1.94)	1.30 (0.51–4.39)	**0.026**^**b**^
LDL-C (mmol/L)	2.93 (1.24–10.12)	2.67 (1.08–4.78)	2.78 (0.79–6.38)	0.169^b^
LDL1-C (mmol/L)	24 (0–75)	21 (2–65)	21 (0–68)	0.203^b^
LDL2-C (mmol/L)	30 (0–62)	28.5 (7–64)	30 (0–82)	0.802^b^
LDL3-C (mmol/L)	14 (0–34)	12 (2–29)	17 (0–73)	**0.009**^**b**^
LDL4-C (mmol/L)	2 (0–26)	2 (0–21)	4 (0–38)	**0.028**^**b**^
LDL5-C (mmol/L)	0 (0–30)	0 (0–4)	0 (0–17)	**0.012**^**b**^
sdLDL-C (mmol/L)	16 (0–84)	16 (3–53)	21 (0–86)	**0.005**^**b**^
IMT (mm)	1.2 (1.0–2.0)	2.4 (1.5–4.5)	2.2 (1.5–6.0)	** < 0.0001**^**b**^

The values in bold mean statistically significant

^a^Fisher’s exact test

^b^ Kruskal–Wallis *H* test

Furthermore, we assessed whether sdLDL-C could be an independent risk factor for individuals with subclinical atherosclerosis. After adjustment for traditional risk factors including age, gender and blood glucose, it was found that sdLDL-C was an independent risk factor of the presence of CAP in subjects with abnormal IMT by multivariable regression analysis (OR = 8.15, 95% CI: 1.52–43.87, *p* = 0.015, Table 4. Besides, systolic pressure and age were also predictors for the presence of CAP.

**Table 4:** Multivariate logistic regression analysis of the independent correlation between sdLDL-C and CAP presence

Variables	Univariate			Multivariate		
	OR	95% CI	*P* value	OR	95% CI	*p* value
Gender (female)	0.56	0.28–1.13	0.107			
Age	16.17	2.54–102.60	**0.003**	25.39	4.63–139.31	** < 0.0001**
BMI	0.24	0.02–2.27	0.212			
Smoking	0.97	0.44–2.13	0.935			
Hypertension history	1.61	0.71–3.64	0.256			
Diastolic pressure	0.29	0.01–9.73	0.486			
Systolic pressure	34.37	2.85–414.89	**0.005**	24.12	3.91–148.88	**0.001**
Blood glucose	4.53	0.18–116.34	0.362			
Total cholesterol	0.49	0.03–9.00	0.632			
Triglyceride	0.77	0.03–20.43	0.876			
HDL-C	0.08	0.001–5.06	0.232			
LDL1-C	1.95	0.14–27.41	0.619			
LDL2-C	1.26	0.08–18.50	0.867			
sdLDL-C	12.22	1.18–126.37	**0.036**	8.15	1.52–43.87	**0.015**

The values in bold mean statistically significant

## Discussion

CVD is giving a heavy burden to the patients and society worldwide. Population screening to find cases with asymptomatic atherosclerosis is one of the effective ways to reduce cardiovascular mortality and morbidity. Although, LDL-C level has been considered to be a useful marker to predict CVD. Increasingly evidence showed that a portion of the population with CVD has normal LDL-C [28]. Thus, current research began to focus on exploring better lipid biomarkers for CVD. For instance, proprotein convertase subtilisin/kexin type 9 (PCSK9), a serine protease, was found playing a crucial role in the regulation of plasma LDL-C concentration by inducing LDL receptor degradation [29]. In addition, lipoprotein(a) [Lp(a)] is another established CVD risk factor, and LDL-C contains a contribution from the cholesterol in Lp(a). Previous study indicates that it has causal association with atherosclerosis due to the proatherogenic LDL-like properties and the prothrombotic plasminogen-like activity of apolipoprotein(a) [apo(a)] [30]. Meanwhile, according to available evidence, sdLDL-C levels have been considered as a potential marker for monitoring CVD and early diagnosis of atherosclerosis [31]. As yet, studies investigating the relationship between asymptomatic atherosclerosis and levels of sdLDL-C are relatively sparse.

In this retrospective study, we reported that sdLDL-C levels were significantly higher in subjects with abnormal IMT (IMT > 1 mm), compared with control group (IMT ≤ 1 mm). And sdLDL-C levels were significantly positively correlated with IMT value and presence of carotid plaque in the subjects with abnormal IMT. In addition, the results suggested that sdLDL-C may contribute to unstable plaque. Notably, sdLDL-C was an independent risk factor of the occurrence of CAP in the Chinese subjects with abnormal IMT.

Despite great effort on the diagnosis and treatment of symptomatic atherosclerosis, screening methods for early detection and treatment of asymptomatic coronary artery disease are still a challenge. CA-IMT and plaques measured by ultrasound are key indicators for carotid atherosclerosis [32]. Nevertheless, carotid ultrasonographic examination is not a routine test for annual health check-up, especially in rural area with limited health care systems. The polyacrylamide gel (PAG) based system of the electrophoretic separation of human plasma lipoproteins enables quick identification and quantitative assessment of the atherogenic lipoproteins, especially the small dense LDL-C. In the present study, we used this FDA-cleared method to analyze the plasma sdLDL-C levels. We found that sdLDL-C positively associated with IMT value and presence of carotid plaque in the subjects with abnormal IMT. And through multivariable regression analysis, sdLDL-C was an independent risk factor of the occurrence of CAP. Our findings indicated that sdLDL-C detected by LipoPrint system could be an alternative way to identify individuals with subclinical atherosclerosis.

LDL cholesterol is a significant risk factor for the development of CVD by evidence from observational studies and clinical trials [33–35]. However, it is worth noting that there are no significant differences in LDL-C level between abnormal IMT group and control group, and LDL-C has no significant correlation with presence of CAP in this study. These results may provide evidence to support previous studies that a relatively high proportion of individuals with normal LDL-C level still develop CVD [9, 36]. In stark contrast, sdLDL has been considered to have more atherogenic effect than other LDL particles due to its increased susceptibility to oxidation, high endothelial permeability, and decreased hepatic LDL receptor affinity [36, 37]. Previous studies demonstrate that sdLDL-C is a new risk factor for cardiovascular events [11, 15, 16, 38]. For instance, one previous study suggested that sdLDL-C concentrations were a better marker for assessment of coronary heart disease (CHD) than total LDL-C [39]. In another study, elevated sdLDL-C concentrations, but not total sdLDL particle concentrations, were reported to be a significant marker of CHD risk in nondiabetic individuals [40]. Moreover, it has been demonstrated that sdLDL-C could predict the CHD risk even in patients considered to be at low cardiovascular risk based on their LDL-C levels in a large prospective study including 11,419 individuals [38]. Meanwhile, a study on the correlation between sdLDL-C and CA-IMT in a healthy Chinese population showed that CA-IMT was significantly associated with sdLDL-C, even being adjusted by traditional CVD risk factors such as higher age, male sex, and other traditional CVD risk factors [41]. These studies combined with our findings indicate that sdLDL-C has an important role in the early development of atherosclerosis, and sdLDL-C could be an additional marker for heart disease early screening in the health check-up. Furthermore, reducing the cholesterol content of LDL-C has been the mainstay of atherosclerosis prevention, but accumulating evidence shows that patients who are treated with lipid-lowering medications and reached a targeted LDL-C level are still at risk of mortality and recurrent cardiovascular and cerebrovascular events [9, 36]. Previous studies and our results imply that not only the total LDL-C level, but also the sdLDL-C levels should be drew attention in the prevention and treatment for CVD.

Carotid ultrasound is a widely-used imaging technique for the assessment of cardiovascular. However, given that the price of ultrasound assay is not cheap compared with blood routine test, and the measurement needs to be performed by a trained and experienced ultrasound technologist. Normally, it is not a routine test for annual health check-up, especially in the low-income rural areas. Moreover, patients should be positioned lying face-up on an examination table for ultrasound, which may limit the results for those with respiratory disease unable to lie flat or those with arthritis incapable of rotating the head. The plasma sdLDL-C assessment is performed by the LipoPrint system. It is easy and simple to handle by technologist in accordance with the manufacturer's instructions. And it is accurate and relatively inexpensive compared to other established methods [42], In addition, it has population-reference values from a normal population as defined by NCEP ATP III guidelines for desirable lipid levels. Combined the results of this study with other previous evidences, sdLDL-C assay may be an alternative way to the asymptomatic atherosclerosis screening, especially for those in areas that lack medical resources.

ertain limitations apply to the present study. First, this was a single-centred, retrospective study. Only Han Chinese in the Kaifeng area were involved in this study. Secondary, the effect of other risk factors such as smoking, drinking, and family history of CVD were not considered in this study. Third, due to the lack of data on sdLDL-C and CVD outcome at follow-up, the results of our study only reflect the relationship of sdLDL-C to IMT and CAP in individuals with abnormal IMT. Further prospective study may better uncover the role of sdLDL on the CVD occurrence in the individuals with clinically silent atherosclerosis.

## Conclusion

In this study, we investigated the association of sdLDL-C with CA-IMT and carotid plaque in individuals with clinically silent atherosclerosis. It was found that IMT was significantly positively correlated with the level of sdLDL-C, instead of LDL-C. The present study suggests that sdLDL-C is an independent risk factor of the occurrence of CAP in the Chinese subjects with abnormal IMT, and sdLDL-C may contribute to unstable plaque. Our findings provide supporting evidence that besides the routine lipid profile, additional blood analytes such as sdLDL-C should be adopted in the health screenings, which may improve CVD prognostication and help guide the preventive treatments.

## Data Availability

All the necessary materials can be found in the text or supplementary materials. Due to the privacy policy, the confidential data materials could only be obtained with the permission of the corresponding authors.

## Abbreviations

CA-IMT: Carotid artery intima-media thickness
CAP: Carotid plaque
CCAs: Common carotid arteries
CHD: Coronary heart disease
CVD: Cardiovascular disease
HDL: High-density lipoprotein
LDL: Low-density lipoprotein
ICA: Internal carotid artery
Lp(a): Lipoprotein(a)
sdLDL-C: Small dense low-density lipoprotein cholesterol
PAG: Polyacrylamide gel
